# Gesture Prediction Using Wearable Sensing Systems with Neural Networks for Temporal Data Analysis

**DOI:** 10.3390/s19030710

**Published:** 2019-02-09

**Authors:** Takahiro Kanokoda, Yuki Kushitani, Moe Shimada, Jun-ichi Shirakashi

**Affiliations:** Department of Electrical and Electronic Engineering, Tokyo University of Agriculture and Technology, 2-24-16 Nakacho, Koganei, Tokyo 184-8588, Japan; s176397r@st.go.tuat.ac.jp (T.K.); s185561y@st.go.tuat.ac.jp (Y.K.); s159177q@st.go.tuat.ac.jp (M.S.)

**Keywords:** data gloves, thin graphite films, gesture prediction, artificial neural networks

## Abstract

A human gesture prediction system can be used to estimate human gestures in advance of the actual action to reduce delays in interactive systems. Hand gestures are particularly necessary for human–computer interaction. Therefore, the gesture prediction system must be able to capture hand movements that are both complex and quick. We have already reported a method that allows strain sensors and wearable devices to be fabricated in a simple and easy manner using pyrolytic graphite sheets (PGSs). The wearable electronics could detect various types of human gestures with high sensitivity, high durability, and fast response. In this study, we demonstrated hand gesture prediction by artificial neural networks (ANNs) using gesture data obtained from data gloves based on PGSs. Our experiments entailed measuring the hand gestures of subjects for learning purposes and we used these data to create four-layered ANNs, which enabled the proposed system to successfully predict hand gestures in real time. A comparison of the proposed method with other algorithms using temporal data analysis suggested that the hand gesture prediction system using ANNs would be able to forecast various types of hand gestures using resistance data obtained from wearable devices based on PGSs.

## 1. Introduction

The recognition of human gestures with the aid of wearable devices has found widespread application in modern manufacturing systems [1,2] and smart assistive technologies [3,4,5,6]. For these applications, it is desirable to detect a human gesture as early as possible [7], to make the human–computer interaction more natural, e.g., deploying a robot to help an elderly person stand up before he/she is upright and is at risk of falling. These human–computer interaction technologies would therefore benefit from human gesture prediction [8]. Among the different human bodily gestures that can be recognized, hand gesture recognition is significant for human–computer interaction because of its extensive application in virtual reality, computer gaming, and sign language recognition [9,10]. Since human gestures involving hand gestures are complicated, capturing the temporal dynamics using highly sensitive wearable devices is fundamental for successful gesture prediction. In recent years, several studies have shown that artificial neural networks (ANNs) are useful for series forecasting [11,12,13,14,15]. Furthermore, time delay neural networks (TDNNs) and recurrent neural networks (RNNs), both of which were proven capable of reliably predicting the future trend of a time series, can also be used to solve these problems [16,17,18,19]. The disadvantage of gesture prediction is that, although it is a highly interesting and important system, the practical application of a system based on wearable devices is considered to be limited by high costs. Therefore, low-cost wearable devices are strongly required.

We previously reported a simple and facile method to fabricate strain sensors and wearable devices using pyrolytic graphite sheets (PGSs) [20]. These PGS sheets are flexible, synthetically produced, uniform, highly oriented, and inexpensive [21]. The sensing mechanism of our strain sensors and wearable devices based on PGSs was explained by modeling over-connected graphene/graphite flakes. The resistances of PGS strain sensors gradually increase as the tensile bending strain increases, and gradually decrease as the compressive bending strain increases. Wearable devices based on PGSs can easily detect various human gestures by using strain sensors to measure the change in resistance. Therefore, we expected it to be possible to build a gesture prediction system using the resistance data generated by the low-cost wearable devices based on PGSs. This report presents the system we developed to demonstrate hand gesture prediction by TDNNs using hand gesture data obtained from data gloves with PGSs. In addition, the accuracy of the prediction model based on ANNs was verified by comparing its performance with that of a multiple linear regression (MLR) model.

## 2. Gesture Prediction Models for Temporal Data Analysis

We introduced various types of ANNs, such as TDNNs and RNNs, for gesture prediction. ANNs are parallel information processing methods for learning complex cause-and-effect relationships between input and output data. In particular, TDNNs and RNNs are well-defined prediction models that provide insight into dynamic functions. In TDNNs, the dynamics of the model are stored using a time-delayed tap, and the nodes are updated by using recursive feedback from the output to the input at the nodal levels for each iterative cycle. TDNNs can only model data within a fixed-size sliding temporal window and are incapable of handling longer-term dependencies [22]. On the other hand, RNNs are dynamic systems that are specifically designed to solve temporal problems by making use of recurrent connections. This mechanism allows RNNs to learn the temporal dynamics of sequential data. The prediction performance of the ANN models was evaluated by comparing their performance with that of a “baseline” prediction model. Traditionally, multiple regression analysis has been used to model the functional relationships between anthropometric measurements. Therefore, our performance assessment evaluated the ANN models against a strong baseline system using the MLR model. MLR is a statistical procedure that is used to predict the values of a response (dependent) variable from a collection of predictor (independent) variable values [23,24]. The ANN models were shown to outperform MLR.

## 3. Experimental Method

### 3.1. Fabrication of Data Gloves Using Thin Graphite Film-Based Strain Sensors

**Figure 1:**
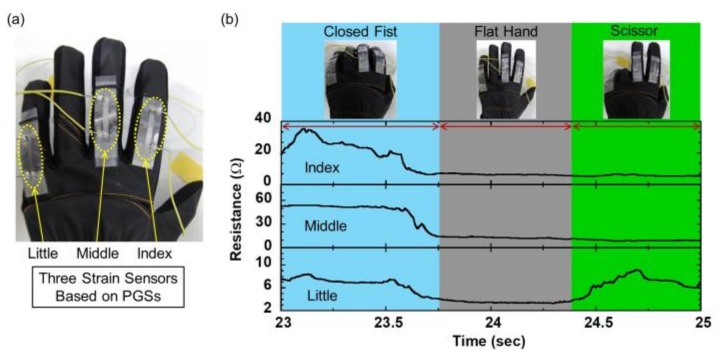
(**a**) Photographic images of data glove based on pyrolytic graphite sheets (PGSs). (**b**) Resistance versus time data for repetitive finger flexions.

First, we fabricated a data glove comprising three independent PGS strain sensors assembled on a commercially available ultrathin glove. These PGS strain sensors were homemade. The strain sensors were positioned over the second joints of the index, middle, and little fingers. In this setting, only these three fingers were considered because the index and middle fingers contribute most to functional gestures such as a closed fist, flat hand, and scissor gesture [25]. The little finger was selected to enable the resistance data to be measured without being influenced by adjacent fingers [26]. This setting is expected to contribute to reduce the complexity of the prediction models. Humans make many different hand gestures with the result that it is difficult to understand them all [27]. This paper focuses on three typical gestures: Closed fist, flat hand, and scissor movement. Figure 1a shows the actual data glove and Figure 1b represents the resistance versus time data for repetitive full finger flexions (i.e., from flat hand to closed fist and back). The flexion of the finger led to an increase in resistance, which was attributed to tensile bending strain. Then, during the motion of extending the finger, the tensile bending strain decreased, and the resistance recovered to the initial state. The sampling rate of each strain sensor was set to 100 Hz. The results indicated that our data glove could easily detect the various hand gestures related to the flexion and extension of each finger. All the gesture measurements reported in this paper were conducted with this data glove.

### 3.2. Procedure for Gesture Prediction System Using TDNN Model

**Figure 2:**
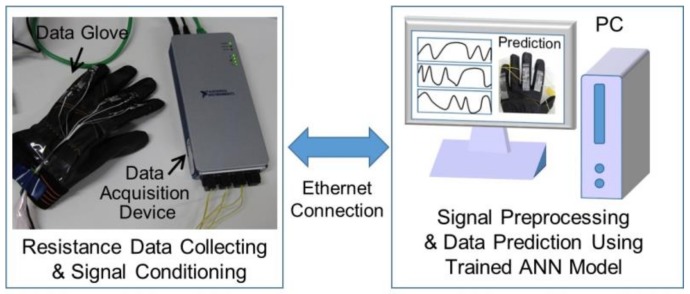
Configuration of the gesture prediction system using the time delay neural network (TDNN) model.

The proposed system consists of the following three main components: The data glove, a data acquisition device, and a PC, as shown in Figure 2. The data glove collects the raw resistance data and the data acquisition device communicates the new resistance data to the PC through an Ethernet connection. The PC predicts the resistance data using the TDNN model. The predicted data are displayed in real time on the PC screen.

**Figure 3:**
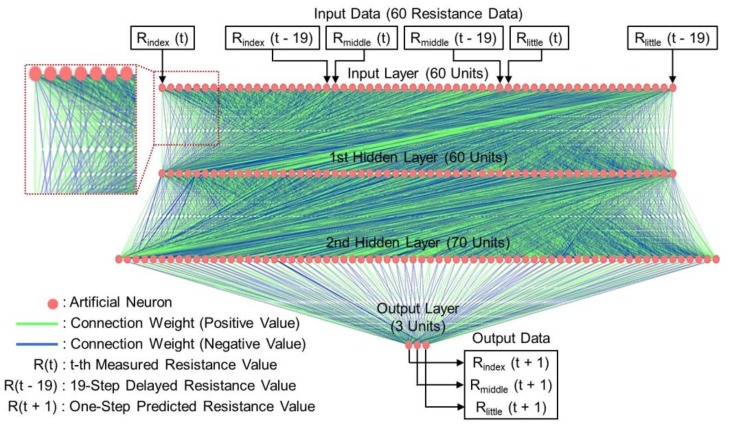
Architecture of the TDNN model with two hidden layers. Green and blue connection lines represent positive and negative relationships, respectively.

The TDNN-based model was established for the prediction of each finger motion to ensure high-precision prediction. Figure 3 exhibits the architecture of the TDNN model, which is constructed with 60 input units, 60 units in the first hidden layer, 70 units in the second hidden layer, and 3 output units. Furthermore, the thickness of the connection line is scaled to the absolute value of the connection weight. The initial values of the connection weights are randomly distributed between +1 and 0. The neurons in the hidden layers and output layer utilize the sigmoid activation function [28].

The temporal dynamics in the sensor signal are learned by training the TDNN model on resistance datasets consisting of data representing the flexion and extension of each finger motion. As shown in Figure 3, the input sequence is a combination of the 20 most recent resistance values obtained from each strain sensor, and the output data are trained to predict the resistance value one point ahead of that of each sensor. The only data pre-processing step the model applied was the standardization of the resistance data of each finger [29]. In the post-processing stage, the predicted resistance data are back-transformed to the actual scale. As training progresses, the connection weights are modified to minimize the errors between the output resistance and the target resistance. To suppress overfitting and enhance the generalization ability of the model, we used max-norm regularization [30]. The TDNN model achieves effective and precise gesture prediction by making use of multi-step-ahead prediction [31]. This prediction method functions as follows: It predicts only one time step ahead, using the estimate of the output of the current prediction, as well as the previous output, as the input to the prediction of the next time step, until the 30-step (300 ms) ahead prediction is made. Thus, the TDNN model could predict hand gestures up to 300 ms ahead.

### 3.3. Architecture of Other Algorithms for Temporal Analysis

**Figure 4:**
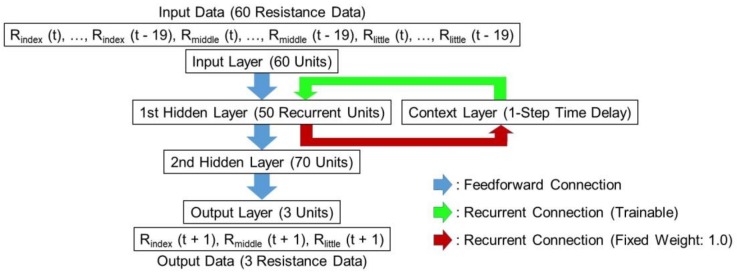
Recurrent neural network (RNN) structure with two hidden layers for hand gesture prediction.

The architecture of the RNN model used in our experiments was configured with 60 input units, 50 units in the first hidden layer, 70 units in the second hidden layer, and 3 output units. In addition, this RNN model also contained a context layer, which is known as a memory layer, in which the output previously generated by the first hidden layer is stored. The output of each hidden unit is copied into a specific unit in the context layer. The output of the context neuron is used as an additional input signal for all the units in the first hidden layer one time step later. Figure 4 presents the RNN model for gesture prediction. Then, the MLR model is used to fit the observed dependent dataset using a linear combination of independent variables. Three MLR models were prepared to predict the resistance of the three strain sensors. The dependent dataset observed with each MLR model contains the resistance values one point ahead of the actual value measured by each strain sensor, and the independent variables are in the same input sequence as those used for the TDNN and RNN models. The respective equations of the three MLR models are as follows:

R_index_ (t + 1) = *a_0_* + *a_1_* R_index_ (t) + *a_2_* R_index_ (t − 1) + … + *a_20_* R_index_ (t − 19)

+ *a_21_* R_middle_ (t) + *a_22_* R_middle_ (t − 1) + … + *a_40_* R_middle_ (t − 19) (1)

+ *a_41_* R_little_ (t) + *a_42_* R_little_ (t − 1) + … + *a_60_* R_little_ (t − 19)

R_middle_ (t + 1) = *b_0_* + *b_1_* R_index_ (t) + *b_2_* R_index_ (t − 1) + … + *b_20_*R_index_ (t − 19)

+ *b_21_*R_middle_ (t) + *b_22_* R_middle_ (t − 1) + … + *b_40_* R_middle_ (t − 19) (2)

+ *b_41_* R_little_ (t) + *b_42_* R_little_ (t − 1) + … + *b_60_*R_little_ (t − 19)

R_little_ (t + 1) = *c_0_* + *c_1_* R_index_ (t) + *c_2_* R_index_ (t − 1) + … + *c_20_* R_index_ (t − 19)

+ *c_21_*R_middle_ (t) + *c_22_* R_middle_ (t − 1) + … +* c_40_* R_middle_ (t − 19) (3)

+ *c_41_*R_little_ (t) + *c_42_*R_little_ (t − 1) + … + *c_60_*R_little_ (t − 19)

where *a, b, *and *c *are coefficients of the first-order linear function.

## 4. Results and Discussion

### 4.1. Network Training for Hand Gesture Prediction

**Figure 5:**
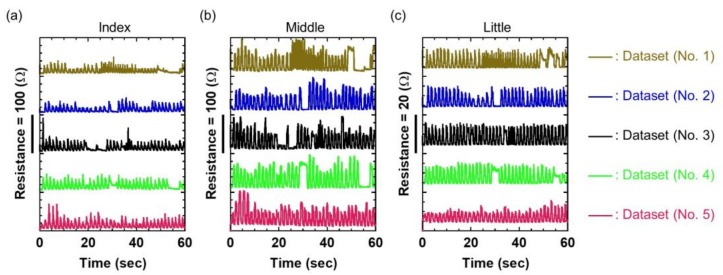
Five training datasets for the (**a**) index finger, (**b**) middle finger, and (**c**) little finger motions, respectively.

**Figure 6:**
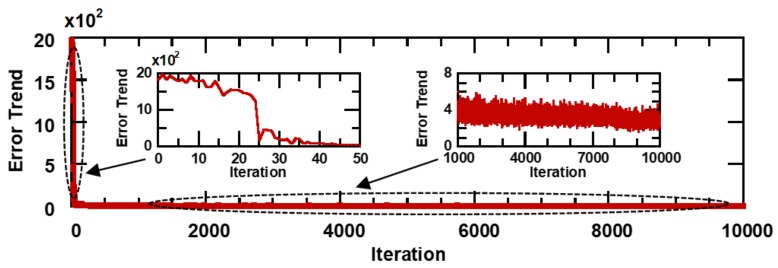
Error curve versus training iteration. The insets represent magnifications of the indicated areas.

**Figure 7:**
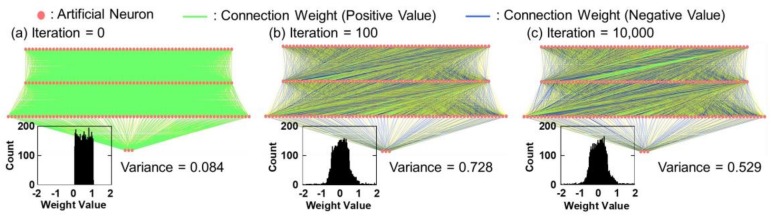
Neural interpretation diagram of TDNN model (**a**) before iteration and then after (**b**) 100 and (**c**) 10,000 iterative computations. The insets illustrate weight distribution of TDNN model.

Training data are important cues for hand gesture prediction. The key requirement is that the gesture dataset should contain various gesture patterns. Eight healthy male participants took part in our data collection experiments. All our resistance datasets contained approximately 50,000 resistance data from each participant. To learn the temporal dynamics of a hand gesture, we collected five datasets, each with a different resistance, consisting of the flexion and extension of each finger motion. Each of these five training datasets, which are summarized in Figure 5, contains 18,000 resistance data. The limited size of the training set may prevent this TDNN framework from obtaining good prediction performances. However, as we can see, the datasets contain data that represent various periodic and non-periodic hand gestures. Therefore, the trained model is expected to predict the correct input–output relationship for the test data. Here, the TDNN model was trained iteratively 10,000 times using back-propagation [28]. Figure 6 shows the normalized training error of the TDNN model. The measured error is one-half of the squared error of all the output units, normalized for the number of training tokens. The enlargements in the insets show that the error curve rapidly diminished during the first 50 iterative computations and then slowly decreased for the remaining iterative computations. This result indicates that the TDNN model may succeed in reaching a suitable weight configuration that minimizes the training error. In addition, we summarized the value of all weights during the training and examined the weight distribution. Figure 7a–c depict the weight connections of the TDNN model as a function of the number of iterative training computations, and the insets illustrate the distribution of a total of 8010 weights. In this training case, because the initial weights were randomly selected between +1 and 0, Figure 7a shows that all weight connections are green lines and the initial weights are uniformly distributed between +1 and 0. Then, as seen in Figure 7b at 100 iterative computations, the weight distribution was centered at zero and the number of weight connections increased rapidly. The converse trend can be observed in Figure 7c after the final iterative computation. In this figure, the weight distribution and connection are almost the same as those in Figure 7b. This indicates that the network has almost finished learning based on the training datasets. The small variance of this weight distribution after training is important for good generalization [32]. This TDNN model thus resulted in a stable and optimized network for gesture data.

### 4.2. Gesture Prediction Using TDNNs in Real Time

**Figure 8:**
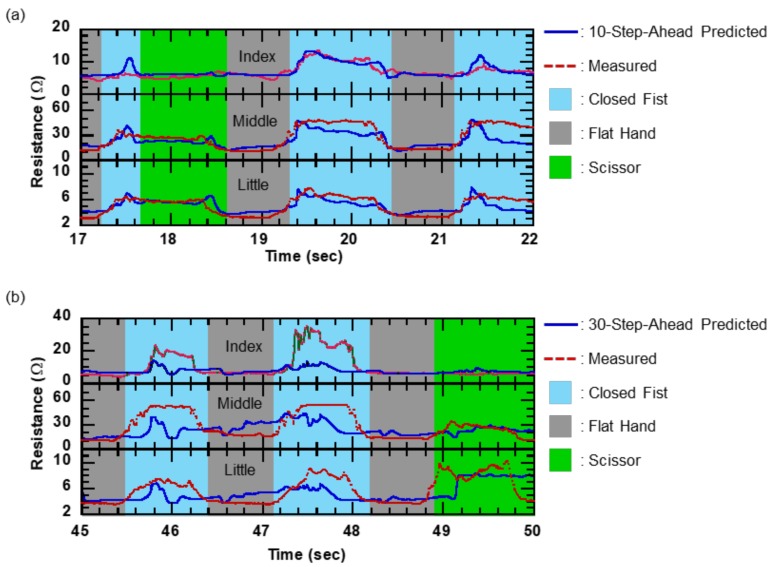
Relation between measured resistance data and (**a**) 10-step-ahead and (**b**) 30-step-ahead predicted resistance using TDNN model. The blue solid and red dashed lines represent predicted and actual data, respectively.

**Figure 9:**
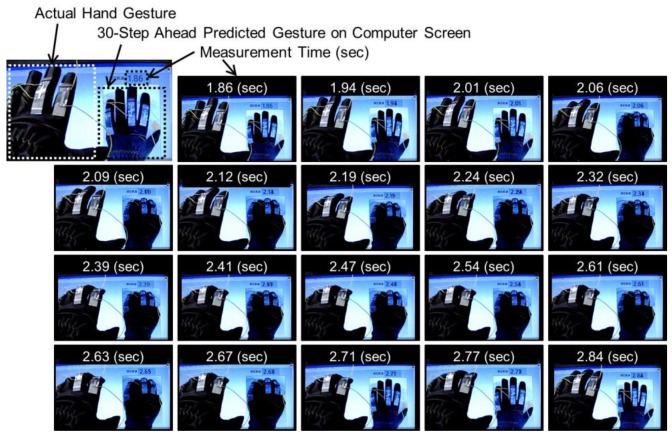
Visualization of the 30-step-ahead predicted gesture obtained from TDNN model.

The predictive ability of the TDNN model was verified by demonstrating the hand gesture prediction capability of the model in real time. Figure 8a,b illustrate the relation between the measured resistance data and the predicted resistance data over 10- and 30-step future time intervals, respectively. The 10-step-ahead predicted data can perfectly detect the trend of actual finger motions and have no obvious lag. Furthermore, the 30-step-ahead predicted data can also capture the feature of flexing finger motion. Therefore, the TDNN model can detect the feature of hand gestures within 300 ms. Moreover, as we can see in Figure 8a,b, the measured resistance data consist of gestures defined by three primitive hand gestures (closed fist, flat hand, and scissor). Although the timing at which the hand gestures change is not linear, the feature of the gestures can be clearly measured by 10-step-ahead predicted data. On the other hand, even in the 30-step-ahead prediction, the TDNN model accurately recognized the resistance data of each hand posture. In addition, Figure 8b shows that the 30-step-ahead prediction could obtain successful gesture predictions within 300 ms, particularly for the flat hand gesture. These results indicate that the predictions obtained are extremely useful, as they predict the major variations in hand gesture without appreciable lag. An example of the visualization of a 30-step-ahead predicted gesture is depicted in Figure 9. In this figure, the predicted hand changed the posture earlier than the actual hand. For example, in the time range from 2.01 to 2.06 s, when the TDNN model predicted a moving from the flat open hand to the closed fist, the predicted gesture was immediately classified as the closed fist. Similarly, in the time range from 2.67 to 2.71 s, when the model predicted that the hand was moved from the closed fist to the flat open hand in future time steps, the model classified the predicted hand gesture as the flat hand. This result indicates the gesture prediction system can contribute to real applications.

We evaluated the predicted signal accuracy by using the mean absolute percentage error (MAPE) [33]. Furthermore, we tested the performance of the gesture prediction by calculating the classification accuracy [34], which is the number of correct decisions divided by the full number of cases. In our study, if the resistance of each finger is greater than the mean of the training data, the finger motion is classified as flexion, otherwise it is classified as extension. The relation between the MAPE and the classification accuracy for each time step is listed in Table 1. The MAPE and the classification accuracy for the 10-step-ahead prediction of each finger were clearly superior to those for the 30-step-ahead prediction of each finger. This difference is mainly attributed to the difficulty associated with accurate long-term prediction. However, the classification accuracy for the 30-step-ahead prediction of the index finger outperformed that for the 10-step-ahead prediction of the little finger. Therefore, successful training of the TDNN model for all fingers would be expected to result in a high probability of the TDNN model making long-term predictions with almost the same accuracy as for short-term prediction. Furthermore, the lowest accuracy of the 30-step-ahead predicted resistances exceeded 50%. As a result, the TDNN model is able to predict hand gesture using resistance data obtained from wearable devices in real time.

### 4.3. Comparison of TDNN Model with Other Algorithms for Temporal Analysis

We verified the adequacy of the prediction performance by comparing the prediction results of the TDNN model with those of the RNN and MLR models using k-fold cross-validation [35]. In this validation, seven validation datasets were prepared from the previously measured datasets to train and test the TDNN model. The validation datasets contain the same data as those that were generated and shown in Figure 5a–c. Then, we evaluated the prediction accuracy of those models using a seven-fold cross-validation method in which each prediction model was trained and tested seven times using the complete validation datasets. In each case, one of the folds is taken as test data and the remaining folds are added to form the training data. Here, the TDNN and RNN models were trained by 1000 epochs, and the MLR model was optimized with a least-squares method [36]. Table 2 and Table 3 contain the results of the 10-step-ahead and 30-step-ahead prediction performance of the models used for the seven-fold cross-validation, respectively. We first compare the results of the TDNN model in Table 2 and Table 3 with those in Table 1. These results demonstrate that the values of MAPE and the classification accuracy are almost the same in both tables. Therefore, the prediction performance of the TDNN model using the seven-fold cross-validation method is almost the same as that of the TDNN model used for real-time gesture prediction. This suggests that the TDNN model delivers accurate and stable prediction performance for various hand gestures. Subsequently, the prediction results of the TDNN model were compared with those of the RNN and MLR models. As seen in Table 2 and Table 3, the TDNN and RNN models outperformed the MLR model. The results in Table 3 indicate that the average MAPE achieved by the RNN model is approximately one quarter of the score obtained by the MLR model. Moreover, the results in Table 2 and Table 3 clearly show that the RNN model is more suitable than the TDNN model for hand gesture prediction. In particular, with the 30-step-ahead prediction, the RNN model outperformed the TDNN model in each respective prediction. These results confirm that the RNN model is suitable for multi-step-ahead prediction. The superior prediction performance of the RNN model compared to the feedforward neural network model can be understood by considering that recurrent connections provide higher nonlinearity [37]. Based on these results, the ANN models have been shown to be effective for predicting hand gestures. In addition, because the prediction accuracy can be improved by optimizing the structure of the TDNN and RNN models, these models are considered to have the capability to predict hand gestures even further ahead in time.

## 5. Conclusions

In the study presented in this paper, we investigated a hand gesture prediction system using hand gesture data obtained in real time from data gloves equipped with PGSs. The low-cost data glove incorporating the PGSs was fabricated by using a simple and facile method, and could accurately detect quick hand gestures in combination with the TDNN model. To demonstrate the potential of the hand gesture prediction system, we computed the 10-step (100 ms) and 30-step (300 ms) ahead predicted resistance in real time. The predicted resistance can determine the expected trend of an actual hand gesture without any obvious lag. As a result, the prediction system is able to successfully predict the hand gesture. Moreover, we measured the unbiased prediction accuracy of the TDNN, RNN, and MLR models by evaluating the performance of these models using a seven-fold cross-validation procedure. A comparison of the results of the TDNN model indicated that the impact of using different datasets for training and testing purposes was negligibly small. This confirmed the superior performance of the TDNN model and its ability to predict various hand gestures successfully. In addition, the results showed that the TDNN and RNN models are more suitable than the MLR model, and that the RNN model delivers the best prediction performance overall. These results suggest that the proposed hand gesture prediction system based on an ANN with PGSs holds immense potential for further development of existing hand gesture recognition techniques, especially for Ambient-Assisted Living (AAL) applications. This work is a pilot and feasibility work with the limited size of the dataset. Therefore, future research will seek to further validate the proposed framework in a larger dataset. Moreover, we plan to extend the structure of ANN models to enable them to accept and process various types of input data, such as the resistance data in combination with the bending angle of each finger.

## Figures and Tables

**Table 1:** Mean absolute percentage error (MAPE) versus classification accuracy based on 4-layer TDNN model, for each time step.

	MAPE (%)				Classification Accuracy (%)			
Prediction	Index	Middle	Little	Average	Index	Middle	Little	Average
10-Step-Ahead	17.2	29.8	16.0	21.0	89.7	88.4	75.8	84.6
30-Step-Ahead	30.2	56.3	29.9	38.8	79.6	50.2	53.6	61.1

**Table 2:** 10-step-ahead prediction performance of seven-fold cross-validation for the three models that were evaluated.

	MAPE (%)				Classification Accuracy (%)			
Model	Index	Middle	Little	Average	Index	Middle	Little	Average
TDNN	21.2	26.2	12.7	20.0	89.6	90.0	91.6	90.4
RNN	19.3	29.8	10.6	19.9	90.6	89.6	92.3	90.8
MLR	14.0	37.5	34.9	28.8	89.8	75.8	60.7	75.4

**Table 3:** 30-step-ahead prediction performance of seven-fold cross-validation for the three models that were evaluated.

	MAPE (%)				Classification Accuracy (%)			
Model	Index	Middle	Little	Average	Index	Middle	Little	Average
TDNN	42.8	58.7	30.2	43.9	74.1	70.3	72.8	72.4
RNN	35.4	52.3	24.1	37.3	75.2	72.1	74.7	74.0
MLR	39.3	50.0	381.2	156.8	70.8	66.8	42.2	59.9
